# Current approaches to identify sections within clinical narratives from electronic health records: a systematic review

**DOI:** 10.1186/s12874-019-0792-y

**Published:** 2019-07-18

**Authors:** Alexandra Pomares-Quimbaya, Markus Kreuzthaler, Stefan Schulz

**Affiliations:** 1 Pontificia Universidad Javeriana, Cra. 7 No 40-62, Bogotá, 110231 Colombia; 2 Institute for Medical Informatics, Statistics and Documentation, Medical University of Graz, Auenbruggerplatz 2, Graz, 8036 Austria

**Keywords:** Electronic health record, Machine learning, Natural language processing, Section identification, Clinical narrative, Free text

## Abstract

**Background:**

The identification of sections in narrative content of Electronic Health Records (EHR) has demonstrated to improve the performance of clinical extraction tasks; however, there is not yet a shared understanding of the concept and its existing methods. The objective is to report the results of a systematic review concerning approaches aimed at identifying sections in narrative content of EHR, using both automatic or semi-automatic methods.

**Methods:**

This review includes articles from the databases: SCOPUS, Web of Science and PubMed (from January 1994 to September 2018). The selection of studies was done using predefined eligibility criteria and applying the PRISMA recommendations. Search criteria were elaborated by using an iterative and collaborative keyword enrichment.

**Results:**

Following the eligibility criteria, 39 studies were selected for analysis. The section identification approaches proposed by these studies vary greatly depending on the kind of narrative, the type of section, and the application. We observed that 57% of them proposed formal methods for identifying sections and 43% adapted a previously created method. Seventy-eight percent were intended for English texts and 41% for discharge summaries. Studies that are able to identify explicit (with headings) and implicit sections correspond to 46%. Regarding the level of granularity, 54% of the studies are able to identify sections, but not subsections. From the technical point of view, the methods can be classified into rule-based methods (59%), machine learning methods (22%) and a combination of both (19%). Hybrid methods showed better results than those relying on pure machine learning approaches, but lower than rule-based methods; however, their scope was more ambitious than the latter ones. Despite all the promising performance results, very few studies reported tests under a formal setup. Almost all the studies relied on custom dictionaries; however, they used them in conjunction with a controlled terminology, most commonly the UMLSⓇ metathesaurus.

**Conclusions:**

Identification of sections in EHR narratives is gaining popularity for improving clinical extraction projects. This study enabled the community working on clinical NLP to gain a formal analysis of this task, including the most successful ways to perform it.

## Background

The wide adoption of health information systems has produced billions of Electronic Health Records (EHR) all over the world [32]. Structured fields and narrative entries in EHR provide highly valuable information to support patient care, which is the primary use of EHR. Furthermore, at large scale, EHR are an invaluable source of information for research and analysis in scenarios where health care experiences for individuals can be used to improve the understanding of health care systems and support public health [47]. These and other activities beyond data support for direct health care delivery are known as secondary use of EHR.

Unfortunately, secondary use of EHR content is not a straightforward task due to the need to deal with its hybrid structure, ranging from coded or structured data, over free-text database entries, entire documents, and let alone multimedia content. EHR systems that include solely coded data provide a great support for billing, quality assurance or decision support, but they have significant limitations, including the difficulty identifying medically relevant aspects resulting in a loss of information [22], which has a fundamental influence on medical decision making and acting. The fact that the communication of humans, and therefore of health professionals, chiefly relies on unconstrained natural language, makes it unrealistic to enforce structured and coded representation of each and every data item in a record. Thus, EHR are very likely to contain narrative content.

Regarding the internal structure of health care documentation, it typically follows the guidelines for interactions between health care professionals and patients (e.g. SOAP (Subjective, Objective, Assessment, Plan), APIE (Assessment, Plan, Implementation, and Evaluation) [19, 23, 63]). In many cases, these guidelines are better addressed by free text, because of their familiarity, ease of use and freedom to express anything the healthcare provider wishes. The automated analysis of clinical texts is, however, hampered by a multitude of factors, e.g., ambiguity, diversity of formats, brevity, careless writing, redundancies, complex longitudinal information [64]. These factors are predominant to a greater or lesser extent, depending on the production process of the clinical texts, especially the editing, formatting and auditing tools provided by EHR systems. There are systems that allow their production without any constraints, others impose predefined fixed templates and rigorous auditing tools, others allow pre-formulated templates to be edited by the author, with possible major impact on the semantics of the underlying information model.

With the aim of extracting structured information from clinical narratives by automatic or semi-automatic methods and systematically enhancing them for secondary use, different strategies have been proposed as part of the initiatives of Clinical Information Extraction [61]. These tasks apply Natural Language Processing (NLP) techniques and Machine Learning (ML) algorithms in order to recognize references to different well-known entity types in the clinical domain, such as medical problems, tests, allergies, risks, adverse events, medications, and treatments. This requires addressing the complexity of natural language, which involves: negated expressions [44], coreferences (different linguistic expressions that refer to the same real-world entity) [66], misspellings [26, 43], different language structures (i.e. English vs. Finnish vs. Korean) [53], acronym or abbreviation detection, expansion and disambiguation [24], and anaphoric relations [4], among others.

One option towards addressing the aforementioned problems is adding some structure to clinical narratives through the identification and interpretation of sections. This task, called from now on **Section Identification**, is defined as detecting the boundaries of text sections and adding semantic annotations. We define a **section** as a text segment that groups together consecutive clauses, phrases or sentences that share the description of one dimension of a patient, patient’s interaction or clinical findings. A section can be marked explicitly, through structural demarcations (headings or subheadings), or can exist implicitly. The main assumption for making this identification is that presumably unstructured texts, actually have an explicit (i.e. marked by headings or using predefined section delimiters) or implicit structure defined by clinical authors.

Even though section identification has demonstrated to improve the performance in clinical extraction tasks such as entity recognition [28], abbreviation resolution [67], cohort retrieval [14] and temporal relation extraction [25], there is not yet a shared understanding regarding the very concept and its use within an analysis pipeline for clinical narratives. Indeed, authors reported different approaches regarding the section granularity; for instance, some studies required the identification of general sections such as *Chief Complaint*, *Physical Exam* or *Assessment* (e.g. [7, 29]); others identified general sections and specific subsections within them as *Gynecological History* or *Allergies* within the *Medical History* (e.g. [9, 58]). In the same way, their methods work under different assumptions (e.g. the narrative must contain headings, the narrative must include formatting information), and the obtained results vary significantly, from formal section annotation for each line of the narrative to the detection of standard headings that may denote the beginning of a section.

This review aims to contribute to a shared understanding of this concept by answering the main research questions:

1. What are the characteristics of the studies that propose or include a strategy for section identification,
2. What are the methods used for the identification of implicit or explicit sections,
3. In which application scenarios section identification has been used,
4. In which contexts section identification has demonstrated good performance,
5. Which terminology systems have been used for characterizing sections.

In order to answer these questions, we carried out a systematic review of available section identification approaches, including the methods, the assumptions they made, the kind of *clinical narrative* they processed, and the terminologies employed.

In the remainder of this article, we use the term *clinical narrative* as a report-style free-text used for clinical documentation. Clinical narratives can occur as clinical notes (e.g. progress notes) in free-text fields in EHR databases, but also as fully-fledged documents (e.g. findings reports, discharge letters).

## Method

### Section identification task: relevant keyword terms

Even though section identification in clinical narratives has been described in several studies, there is no consensus about the terms used to refer to it. Therefore, during the first phase of this review, it was necessary to iteratively identify the list of relevant terms that allowed us to perform a complete literature search.

The search strategy was divided into three parts, each of them associated with one aspect: what should be identified, from where it should be identified and how it should be identified. The first aspect was subdivided into two parts: what is the intended action and what is the object of that action. The resulted terms related to the action were: “labeling”, “tagging”, “detection”, “annotation”, “mapping”, “identification”, “segmentation”, “classification”, “decomposing”, “evaluating”, “assessment”, “validation”, and several variations, for instance “annotated”, “label”, “mapped”, among others. The terms employed to describe the object were: “zone”, “segment”, “section”, “header”, “paragraph”, “sector”, and their variations as “subsections”, “headings”, among others.

The purpose of the second aspect was to define the scope of the analysis, because this review is intended to analyze studies exclusively in clinical narratives, and not in other types of documents, especially not scientific abstracts. This term comprises those core parts of the medical record that are relevant for the provision of care, e.g., medical history, nursing notes, reports of special procedures, and summaries. Thus, we included the terms or acronyms: “EHR”, “EMR”, “clinical record”, “medical record”, “health record”, “patient record”, “clinical text”, “medical narrative”, “clinical narrative”, “clinical report”, “clinical note”, “clinical document”, and their plural variations.

Considering the scope of this review, we also included a set of terms that allowed us to identify studies that involved *automatic or semi-automatic processes*, resulting in the inclusion of the following terms: “language processing”, “NLP”, “machine learning”, “mining”, “data analytics”, “corpus”, “text analysis”, “medical informatics”, “deep learning”, “big data”, “artificial intelligence”, “retrieval”, “automated”, “information extraction”, “pattern recognition”, and variations thereof.

The explicit inclusion of term variations in each part of the search expression was important, because the query mechanism of some literature databases is not tolerant regarding morphology (e.g. stemming or lemmatization). The integration of the final set of terms was made using the OR operator within each query aspect and the AND operator between the aspects.

### Eligibility criteria and information sources

Considering our research questions, the eligibility criteria comprised two inclusion criteria: **IC1**: The study proposes, uses or evaluates a section identification task on EHR narratives. **IC2**: The section identification task is explained and, it is done using an automatic or semiautomatic method.

IC1 allows us to include three types of studies: 1. Studies whose main goal is proposing a section identification method. 2. Studies that use a section identification method in order to achieve another objective of greater scope, such as named entity recognition or de-identification. 3. Studies that evaluate a section identification method proposed in another work or using a specific software tool.

IC2 allows us to include two types of studies: 1. Studies that elucidate the method they propose. 2. Studies that apply and explain a method proposed by others.

Studies from 1994 until 2018 in any language were eligible. 1994 was chosen because, after this year, the popularity of the internet promoted the creation of automated information systems, including EHR systems. These studies were identified in three well-known databases: SCOPUS, Web of Science and PubMed using the search query previously defined.

### Selection process

**Fig. 1:**
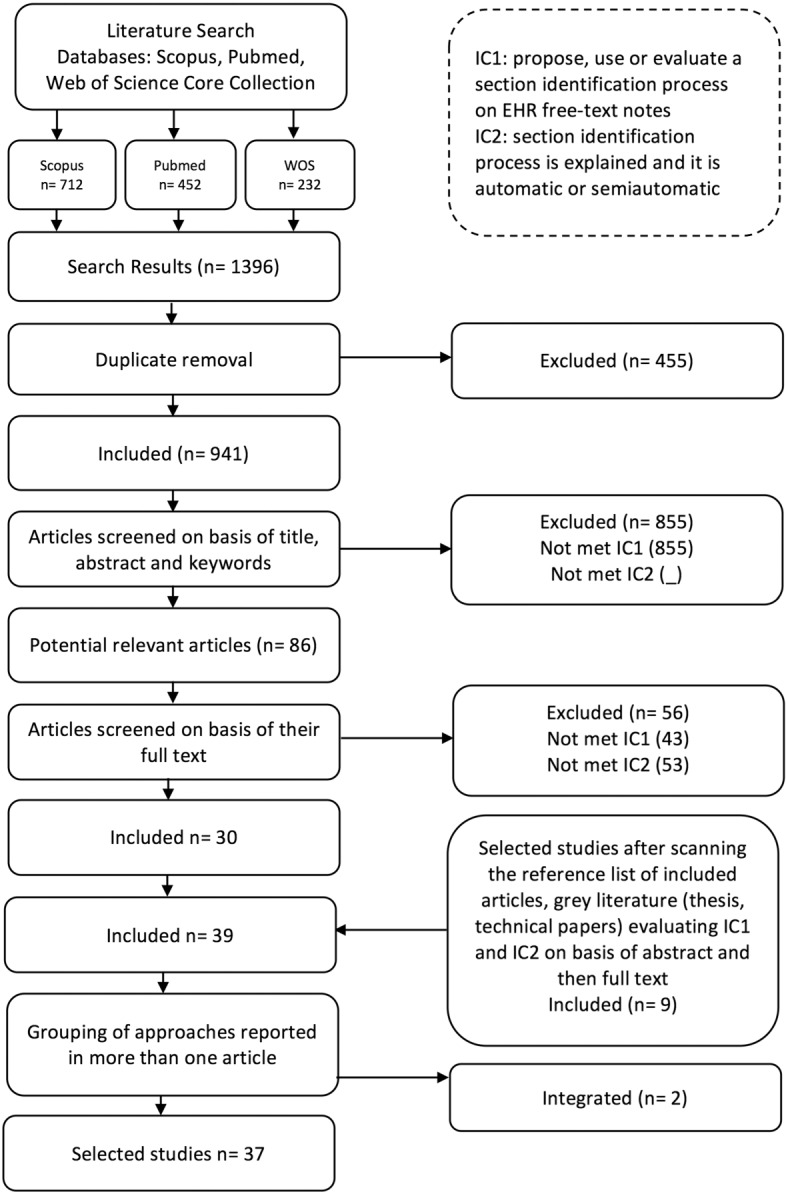
Selection method

Our selection process is depicted in Fig. 1. The literature databases provided a total of 1396 studies. Results were exported to a CSV (Comma-Separated Values) file and imported into Microsoft Excel, where duplicates were removed, thus obtaining 941 studies. For each of them, titles, abstracts, and keywords were independently examined by two of the authors of this review in order to determine if they met the specified eligibility criteria. As a result, 86 articles remained for further analysis. These articles were completely read by the first author, discarding 56 of them for not meeting the eligibility criteria. Another author of this article verified again the eligibility criteria in the 56 discarded studies by reading their method section (or equivalent) and their conclusions. A total of 30 articles were therefore included in the review. Once these 30 studies were examined in depth, all the authors of this review examined their reference lists for possibly relevant articles.

In addition, the authors checked in the source databases PubMed and Scopus the list of similar or related articles to the relevant ones. Finally, one of the authors searched on Google Scholar for another set of possibly relevant articles. After verifying that the obtained articles were different from those previously reviewed, the final list included nine (9) additional relevant studies. Finally, 39 articles were studied in depth. Studies on sentence-level chunking were discarded (e.g. [41]).

### Analysis method

For the analysis, we grouped the studies with the same method, the same focus and with at least one common author, e.g., two of the articles focusing on the SecTag method were put in one group Denny et al. [9, 10], and the works of Chen and Dai were put into another group Dai et al. [7], Chen et al. [5]. The other studies were treated independently. Thus, 37 studies, including the grouped ones were analyzed, considering the main research questions.

## Results

During the analysis we found that each study addressed the section identification task with a different focus; a subset addressed it as part of a comprehensive text analysis pipeline; others addressed it as a primary task that, given its complexity, required a major effort to achieve good results and, as a consequence, some of them produced modular solutions.

As Table 1 presents, 16 (43%) studies have a secondary focus on section identification and 21 (57%) studies considered it a primary task. The article by Denny et al. [10] is the only one whose results were reused in institutions other than the authors’ [34]. Besides, two works reused the section identification algorithms provided by external entities: MedLEE (the Medical Language Extraction and Encoding System) [15] ([60]) and the Apache cTAKES [48] ([30]).

**Table 1:** Profiling of the studies

Reference	Method	Focus	Section	Type	Language	Narrative
Doan et al. [12]	R	S	G&S	B	English	D
Denny et al. [9] and Denny et al. [10]	R	P	G&S	B	English	H,Phy
Denny et al. [11]	R	S	G&S	B	English	D,P
Edinger et al. [14]	R	P	G	E	English	D,R,N
Hsu et al. [18]	R	S	G&S	B	English	Pat,R
Kropf et al. [25]	R	S	G&S	E	German	Pat
Lee and Choi [27]	R	P	G	B	Korean	D
Lin et al. [30]	R	S	G	E	English	C,Pat
Mehrabi et al. [34]	R	S	G&S	E	English	C
Melton et al. [35]	R	P	G&S	E	English	O
Meystre and Haug [36]	R	S	G	E	English	NS
Taira et al. [55]	R	P	G	B	English	R
Phuong and Chau [42]	R	S	G	E	English	D
Rubin and Desser [45]	R	S	G	E	English	R
Schadow and McDonald [49]	R	S	G&S	E	English	Pat
Schuemie et al. [50]	R	P	G&S	E	English	C
Shivade et al. [51]	R	S	G&S	B	English	D,A,L
Singh et al. [52]	R	S	G	E	English	R
Suominen et al. [54]	R	S	G	E	Finish	D,A,P,N
Tran et al. [58]	R	P	G&S	B	English	NS
Wang et al. [60]	R	S	G	E	English	D
Xu et al. [65]	R	S	G	E	English	D
Bramsen et al. [3]	ML	P	G	I	English	D
Deléger and Névéol [8]	ML	S	G	I	French	NS
Haug et al. [17]	ML	P	G	B	English	D,O,C,Pat,R,H,Phy
Jancsary et al. [20] and Jancsary [21]	H	P	G&S	E	German	Dic
Li et al. [29]	ML	P	G	B	English	D,A,C
Lohr et al. [31]	ML	P	G&S	B	German	D
Mowery et al. [39]	ML	P	G&S	B	English	E
Tepper et al. [57]	ML	P	G&S	B	English	D,R
Waranusast et al. [62]	ML	P	G	I	Other	C
Apostolova et al. [1]	H	P	G	B	English	R
Cho et al. [6]	H	P	G&S	B	English	R,U
Dai et al. [7], Chen et al. [5]	H	P	G	B	English	D,O
Ganesan and Subotin [16]	H	P	G	E	English	NS
Ni et al. [40]	H	P	G&S	E	NS	NS
Sadoughi et al. [46]	H	P	G	B	English	Dic

NS=Not Specified; Focus: P=Primary, S=Secondary; Sections: G=General sections, S=General and Subsections; Type: E=Explicit, I=Implicit, B=Both; Narrative: D=Discharge Summaries, A=Admission Notes, P=Progress Notes, O=Operative or Procedure Notes, C=Clinic Visit Notes, E=Emergency Reports, Pat=Pathology Reports, R=Radiology Reports, U=Urology Reports, H=History or Family History, Phy=Physical Exam, N=Nursing Notes, Dic=Medical Dictation, L=Letter of Communication; Method: ML=Machine Learning, R=Rule-based, H=Hybrid

### Profiling of the studies

Studies on section identification vary greatly depending on the kind of narratives and the type of sections they are focused on. This section describes the scope of each study in terms of the kind of section that it is able to recognize, the assumptions it makes during the section identification process, the type of narratives that the study addresses, and the language that it can recognize.

As shown in Table 1, most of the studies (54%) identify general sections, i.e. sections that coarsely refer to the typical steps of a clinical treatment episode, such as *Medical History* or *Medications*. 46% of the studies also detect subsections, e.g., *Allergies* within *Medical history* or *Specimens* within the *Diagnosis* section of a pathology report. Some articles are only concerned in detecting one general section, like Singh et al. [52], who detect the *Impression* section from radiology reports, or Mehrabi et al. [34] who aim at detecting nothing but the *Family history* section and its subsections. Besides section types, the scope of some studies is limited by their methodological assumptions. The most common assumption is that all sections are preceded by sections headings. We refer to this as explicit sections. Forty-six percent of the studies subscribe to this assumption. Other ones detect implicit sections, i.e., sections without headings (8%) or can detect both (46%). Regarding the language of the narratives, English is leading with 78%, with the rest split between German, Korean, Finnish and French. This reveals a large gap regarding solutions tailored to other languages. The type of narrative showed a large diversity, with discharge summaries leading with 41% of the studies, probably because they usually contain an internal structure defined by local or institutional regulations or because they are frequently transferred between practitioners, or institutions. Radiology reports are in second place in 24% of the studies.

Even though the EHR System from which clinical narratives were generated plays a crucial role in their structure, several studies did not report it. However, from the studies that did report the source of the narratives, we could identify that they vary considerably. Some studies used analytical repositories: the Intermountain Healthcare’s Enterprise Data Warehouse (EDW) [17], the VINCI (Veterans Informatics and Computing Infrastructure) database [58], the MARS(Medical ARchival System) repository [39], the data repository of the New York-Presbyterian Hospital [29], and the SAP Business Warehouse [31]. Another group of studies extracted the narratives directly from an EHR or EMR (Electronic Medical Record) system: the Vanderbilt’s EMR system [10], the Seoul National University Hospital EHR [27], and the UW Radiology Information System [57]. Other studies used narratives from speech recognition systems or dictation systems: [1, 20], and [46]. Finally, there is a group of studies that used external sources such as the ones provided by competitions: [7] and [57]; or other open data sources such as the corpus of medical discharge summaries from the online edition of The New England Journal of Medicine [3].

### Methods used for section identification

Current section identification methods can be classified into rule-based (59%), ML (22%) and hybrid (19%) methods. The former ones are based on a set of rules, created by experts or using methods that explicitly describe patterns for detecting section boundaries. These rules are validated with a sample of the available texts, and then applied to new ones. ML methods train a classification model, using a text corpora in which sections were previously identified and annotated (i.e. sections are clearly identified); the generated model is then applied to classify sections in new texts. Hybrid methods take the best of both; rules that detect well-known clues first identify easily detectable sections; this step is then refined using a ML model. Table 1 classifies the studies according to these methods.

#### Rule-based approaches

Studies that use rules range from simple rules that match section headings against target text to sophisticated hierarchical rules. In order to characterize these studies, we use the following dimensions (see Table 2):

**Table 2:** Rule based studies

Reference	Type of rules	Info. required	Generated features	Method
Edinger et al. [14]	E	Flat	NS	R
Ni et al. [40]	E	Flat	NS	H
Phuong and Chau [42]	E	Flat	NS	R
Singh et al. [52]	E	Flat	NS	R
Wang et al. [60]	E	Flat	NS	R
Apostolova et al. [1]	R	Flat	F	H
Chen et al. [5], Dai et al. [7]	R	Flat	F	H
Hsu et al. [18]	R	Flat	F	R
Kropf et al. [25]	R	Flat	NS	R
Lin et al. [30]	R	Flat	NS	R
Melton et al. [35]	R	Flat	F	R
Meystre and Haug [36]	R	Flat	F	R
Rubin and Desser [45]	R	Flat	NS	R
Sadoughi et al. [46]	R	Flat	F	H
Schadow and McDonald [49]	R	Flat	NS	R
Schuemie et al. [50]	R	Do not require	F	R
Taira et al. [55]	R	Flat	F	R
Ganesan and Subotin [16]	E,R	Flat	F	H
Jancsary et al. [20]	E,R	Dictionary	NS	H
Lee and Choi [27]	P	Hierarchy	NS	R
Tran et al. [58]	P	Hierarchy	F,C	R
Suominen et al. [54]	R,P	Flat	F,C	R
Cho et al. [6]	E,R,P	Flat	F,H	H
Denny et al. [9]	E,R,P	Hierarchy	F,H	R
Denny et al. [11]	E,R,P	Hierarchy	F,H	R
Doan et al. [12]	E,R,P	Hierarchy	F,H	R
Mehrabi et al. [34]	E,R,P	Hierarchy	F,H	R
Shivade et al. [51]	E,R,P	Flat	F,C	R
Xu et al. [65]	E,R,P	Hierarchy	F,H	R

NS=Not Specified; Type of Rules: E=Exact matching, R=Regular Expressions, P=Probabilistic rules;Generated Features: F=Formatting features, C= Concept or terms contained, H=Heading Probabilities; Method: R=Rule-based, H=Hybrid

1. **Type of Rules:** A rule is an expression that describes the way things happen in a particular situation. In this sense, rules express general or specific characteristics of these situations. Section identification rules describe how sections of a certain type are usually written.
  - **Exact matching**: This kind of rule identifies the beginning of a section by the appearance of a heading available in the terminology and the ending before the next heading starts.
  - **Regular expressions**: They describe sets of rules using search patterns, which are then matched against the target text. A search pattern specifies the type of text you want to find in the target text using constants and operators. A regular expression may use terminologies as input. For instance, it may express that a heading text (from an existing terminology) followed by a colon marks the beginning of a section.
  - **Probabilistic rules**: Rules that take into account probabilistic data obtained from sample data sets, such as Bayesian word probabilities occurring in each section of a sample text.
  Different types of rules may often be used in the same study. For instance, a method may first find standard headings using an exact matching rule and then identify variations using regular expressions.
2. **Required previous information:**This dimension explores the type of external information required as input by the method. Studies included in this review use:
  - **Flat** dictionaries of headings: These are plain lists that contain possible terms used as headings for each section.
  - **Hierarchical** heading dictionaries: They include a hierarchy of headings, which implies a parent-child relationship that can be exploited to infer knowledge when a heading coincides with a portion of the target text.
  - **Dictionaries** of synonyms and word variants, providing modifications or abbreviations of standard headings. Some of the studies include a dictionary with the most common variations or synonyms for a heading or words contained therein.
3. **Features Generated from Available Sample Texts:** Some studies rely on information extracted from an available sample of texts that can give hints on the existing patterns for different sections. This dimension describes what kind of features are extracted from these texts to be used later as an input for the rules. Common features can be classified in the following groups:
  - **Formatting** features: Represent the shape of a section including the characteristics of its headings (e.g. word count, capitalization), explicit line ending characters (e.g. question marks), the beginning of the lines matching an enumeration pattern, or the typical size of the section.
  - **Concepts/ terms** within each section: Frequent concepts or terms occurring in each section are identified.
  - **Heading** probabilities: Probabilities are assigned to common and uncommon section headings in a corpus; e.g., the probability of each possible section heading occurring in a document within a corpus.
  These features can only be obtained automatically if the sample data set was already annotated with section begin and end tags. Note that feature values may vary according to the corpus, which means that the rules generated using them should be dynamically adapted to new feature values to be used in different contexts (i.e. the typical size of a section may differ between corpora).

The following paragraphs detail the characteristics of section identification studies that use a rule-based approach. Basic approaches rely on the **exact matching** of well-known headings (and some heading variations) within clinical narratives; different studies follow this approach. Wang et al. [60] detect sections in discharge summaries finding a set of predetermined section headings and their target forms. Their work, based on the MedLEEs section identification method [15], demonstrates that selecting information considering the section where it is contained, may improve the detection of drug disease-manifestation related symptoms and drug-drug event relations. Similarly, the work proposed by Edinger et al. [14] improves cohort selection looking for the name of the sections where a specific patient characteristic should be found. Likewise, the study by Singh et al. [52] classifies radiology reports into high and low priority ones by analyzing the *Impression* section, which was detected using hand-crafted rules.

Finally, the study of Phuong and Chau [42] recognizes the beginning of a section looking for the well-known headings or words in order to apply de-identification processes taking into account the section where the possible identifiers are.

The studies above demonstrate that exact matching of heading texts for section identification is very useful to improve the precision of this task. However, if the level of standardization of the headings is low, recall drops due to sections that cannot be detected by this method.

As an evolution of the rules that find the exact words of headings in the text, **regular expressions**, and in general pattern matching, are the most common methods used for section identification. Radiology reports were analyzed using regular expressions in different studies. Taira et al. [55] and Rubin and Desser [45] identified sections in this type of documents using classifiers that include a dictionary of common section labels (e.g. *Clinical History*, *Findings*) and common formatting layouts (e.g. section order, section length, use of colons). Hsu et al. [18] proposed a quality assurance process for evaluating the diagnostic accuracy of radiology reports, which included section analysis. In a first phase, regular expressions identify relevant sections based on headings. In addition, they exploit formatting characteristics such as capitalization, colon use and paragraph breaks for identifying subsections.

Pathology reports sections have also been identified using regular expressions. Schadow and McDonald [49] created regular expressions to find terminology for specimen headings. Kropf et al. [25] improved information retrieval of pathology reports by means of section identification. Basically, the results of queries for certain tumors in pathology reports are contextualized using section-sensitive queries. Other works on pathology reports use systems previously created with this functionality; for example, Lin et al. [30] used CTAKES sectionizer, based on regular expressions.

In order to analyze the use of standard terminology in the sections of operative notes, Melton et al. [35] extracted headings using rules for capitalization, semi-colons, hyphens, and line-breaks. Potential section headings are then mapped to a controlled terminology. This study found that about 20% of the sections were not covered by these controlled terminologies. Several non-matching sections were, however, important elements for operative notes in certain sub-specialties, such as cardiac and transplant surgeries. This demonstrates that regular expressions based on formatting characteristics complement exact matching rules on the detection of headings that do not follow standard terminologies. Besides, the output of this kind of methods can be used to enrich standard terminologies with interface terms (i.e. terms that do not necessarily correspond to standard terminology labels, but which are characteristic for the clinical jargon) that can improve the recall of section identification in not seen texts.

Sections of clinic visit notes have been identified using regular expressions. Schuemie et al. [50] proposed some section detection heuristics, e.g., lines consisting of a sequence of uppercase letters, optionally followed by a colon, mark the beginning of a section, whereas too many words in the heading, explicit line endings like question marks and periods, or enumeration-like characters at the beginning of a line, prevent flagging these lines as section beginning.

Finally, Meystre and Haug [36] used regular expressions for extracting possible sections headings and then, considering the identified sections, they extracted clinical problem mentions to populate a problem list.

In summary, studies have used regular expressions to find headings that do not match with the standard or expected text, but with some variation of it. Besides, this type of rule is often used to recognize when a text corresponds to a heading, or conversely when it does not, analyzing the formatting characteristics (e.g. capitalization, spaces, length).

Complementing the exact matching and the regular expressions for section identification, a significant subset of studies include more sophisticated rules intended to detect not only labeled, but unlabeled sections. These studies typically use **probabilistic information** obtained from a corpus of clinical narratives.

Denny et al. [9] created a section identification system called SecTag that relies on a hierarchical heading terminology. The approach first identifies sentence boundaries and list elements. Within each sentence it detects all candidate section headings by using rules, spelling correction, recognizing word variants and synonyms that map to known terms and, finally, the method detects section boundaries. In case of low certainty of whether a sentence belongs to a section, it uses a statistical model that contains the prior probability of each possible section heading occurring in a document, the probability of each section heading occurring in a specific position, and the probabilistic sequential order for headings appearing in documents, obtained from a sample data set. Using this model, the system applies different rules to maintain or discard the previously detected sections. For instance, it uses the distance of the candidate section heading to other nearby section headings to decide whether the section should be labeled. It includes a set of rules that take into account the Bayesian scores of each detected section, as well as other kinds of inputs like the typical section length, according to the corpus. This proposal demonstrated very good results for section identification in history and physical examination texts and has been re-used in other studies [11, 12, 34].

The study of Shivade et al. [51] includes two strategies; first, a rule-based strategy that relies on information as the length of a string when it is a section heading, the usage of camel case, and the set of commonly used words that constitute a section heading. The second one is focused on implicit sections. Here, the authors identified the sections by the relative frequency of concepts occurring therein. For instance, a medication section that cannot be explicitly identified using headings can be detected through the identification of concepts that correspond to medication names. Similarly, in Suominen et al. [54], a semiautomatic heading identification was applied using, first, regular expressions, and then a content analysis method that allowed for monitoring shifts in content and style.

The study proposed by Tran et al. [58] detected sections using rules for identifying concepts from an ontology. Each section is associated with a class of this ontology, and the properties of each class represent the terms frequently found in each section, their frequency being obtained from sample data. The key point here is that these properties are used to detect implicit sections in new texts.

Other types of works explore section identification from a temporal point of view. Lee and Choi [27] developed a temporal segmentation method for clinical texts, particularly discharge summaries. Hierarchical rules are created manually based on cue phrases that can indicate topical or temporal boundaries in text structures. Temporal segments can describe information about symptoms, clinical tests, diagnoses, medications, treatments, and clinical department/visit information.

As can be seen, studies have used probabilistic rules mainly to deal with implicit sections, or as a mechanism to improve precision and recall of sections identified with other types of rules. Even though these rules may vary in complexity, all of them represent the knowledge acquired from a sample of texts where sections have been previously identified. The availability of this sample is a crucial constraint for using this type of rules.

Finally, some studies that follow a hybrid approach apply rules during their first phases. That is the case of Ni et al. [40], who defined exact matching rules; others used regular expressions to locate headings and boundary markers [1, 7, 20, 46], and [21]; furthermore, one study used probabilistic rules with statistics computed for each kind of narrative (e.g. the section length, the number of sections, and section order) to identify undetected sections [6]. These studies will be discussed in detail later.

#### Machine learning approaches

ML algorithms are increasingly relevant for section identification. Table 3 presents the studies that include a ML method. Each study is described using the following characteristics:

1. **The ML method(s) used**. Note that this review only illustrates the method (aka technique), since most of the studies do not specify the particular implementation or algorithm used.

**Table 3:** Machine learning studies

Reference	ML method	Training and test data set source	Training data set size	Test data set size	Method
Bramsen et al. [3]	AdaBoost	M	60	CV	ML
Haug et al. [17]	Bayesian Network	M	3483	CV	ML
Chen et al. [5], Dai et al. [7]	Conditional Random Fields	M, RB, CO	790	514	H
Deléger and Névéol [8]	Conditional Random Fields	M	100	600	ML
Ni et al. [40]	Conditional Random Fields and Maximum Entropy Classifier	M, AL	NS	NS	H
Jancsary et al. [20]	Conditional Random Fields and Viterbi	M, RB	2340	1003	H
Cho et al. [6]	Expectation Maximization Classifier	M, RB	NS	NS	H
Li et al. [29]	Hidden Markov Model and Viterbi	M, RB	7549	2130	ML
Lohr et al. [31]	Logistic Regression	M	1106	CV	ML
Ganesan and Subotin [16]	Logistic Regression and Viterbi	M, RB	1800	12502	H
Tepper et al. [57]	Maximum Entropy Classifier	M, CO	1365	374	ML
Sadoughi et al. [46]	Neural Network	M, RB	25842	2000	H
Apostolova et al. [1]	Support Vector Machine	M, RB	3000	200	H
Mowery et al. [39]	Support Vector Machine	M	50	CV	ML
Waranusast et al. [62]	Support Vector Machine and KNN	M	10694	CV	ML

NS=Not Specified; Training and Test Data Set Source: M=Manually created, RB=Using a rule-based approach, CO= Using a data set provided by competition organizers, AC= Using an active learning strategy; Test Data Set Size: CV=Cross Validation; Method: ML=Machine Learning, H=Hybrid
2. **Strategy used for training and test data set creation.** “Manually created” data sets means that human experts annotate (or label) the sections or the specific characteristics from the text required for section identification. “Using a rule-based approach or an automated method” means that the study included an automatic phase that obtained the sections using rules. Finally, “Active learning and distant supervision” means the study facilitates the creation of training and test data sets by combining automatic with a manual method in order to reduce the human effort.
3. **The reported size of the training data set.** Although some studies reported their training sets in terms of sentences, sections, or documents, this review unifies the metric as the number of clinical narratives used.
4. **The reported size of the test data set.** It is described using the same unit (i.e. number of documents). “CV” means Cross Validation, i.e., an approach that uses the same data set for training and testing, changing iteratively the part of the data used for either purpose.

Our analysis shows that the most popular ML methods are Conditional Random Fields (CRF) and Support Vector Machine (SVM). The Viterbi algorithm is also very popular to find the optimal section label sequence where section identification is treated as a sequence labeling problem. All these works rely on manually created training and test sets, at least partially.

The size of the training data set differs considerably between studies, ranging from 50 to 25,842 texts. Size variation notably impacted the performance and adaptability of the models on new data sets.

All studies extracted features relevant for section identification. Table 4 assigns the reported features to the following groups:

1. **Lexical features:** word-level features that can be extracted directly from the text. E.g., the entire word, its prefix, its suffix, capitalization (e.g. uppercase, title case), its type (e.g. number, stop word), among others.

**Table 4:** Machine learning features

Reference	Lexical	Syntactical	Semantic	Contextual	Method
Bramsen et al. [3]	U,N	POS	RT,AT,T	LP	ML
Haug et al. [17]	N	NS	NS	NS	ML
Chen et al. [5], Dai et al. [7]	C,A	Pun	ST	WL	H
Deléger and Névéol [8]	NS	NS	NS	NS	ML
Ni et al. [40]	NS	NS	NS	NS	H
Jancsary et al. [20]	N	POS,Pun	ST	LP	H
Cho et al. [6]	NS	NS	ST	SS,OS	H
Li et al. [29]	N	NS	NS	SB	ML
Lohr et al. [31]	U	NS	NS	NS	ML
Ganesan and Subotin [16]	U,N,C	NS	ST	LP,LL,LC,CC	H
Tepper et al. [57]	U,C	Nu	NS	LP,WL,SS,SB	ML
Sadoughi et al. [46]	NS	NS	NS	SB	H
Apostolova et al. [1]	N,C	Pun	ST	LP,WL,SB	H
Mowery et al. [39]	U,N	POS,VT	ST,DI,MN	LP,LL,SB	ML
Waranusast et al. [62]	NS	NS	NS	SB	ML

NS=Not Specified; Lexical: U=Unigram, N=N-gram, C= Capitalized, A=Affixes ; Syntactical: POS=Word Part of Speech, VT=Verb Tense, Pun=Punctuation, Nu=contains of begins with a number; Semantic: ST=Semantic Type (e.g. UMLS, LOINC), DI=De-identification tag, MN=Meaning of the number(e.g. phone, dosis), RT=is it a relative temporal word (e.g. later, next, until), AT=is it an absolute temporal word (e.g. am, pm), T=Topic of the section; Contextual: LP=Line position in the document, LL=Length of a line, WL=White lines before and after a line, LC=Length change from one line to another, SS=Section size, SB=Previous and following section boundaries, OS=Order of sections, CC= Capital and colon use; Method: ML=Machine Learning, H=Hybrid
2. **Syntactical features**: represent the sentence structure, e.g. grammatical roles and part of speech.
3. **Semantic features**: represent the meaning of words and terms in a sentence. Most of them refer to a custom dictionary or a controlled terminology.
4. **Contextual features**: describe relative or absolute characteristics of a line or a section within the clinical narrative. For instance, the position of the section in the document and layout characteristics.

All studies that applied ML are methodologically similar, but each of them has certain restrictions according to the type of sections it seeks to identify. For instance, the approach proposed by Bramsen et al. [3] explores sections from a temporal perspective. They identify a new section when there is an abrupt change in the temporal focus of the text. E.g., a patient’s admission has a different temporal focus compared to a previous hospital visit. Using the training data, the method extracts boundaries that delineate temporal transitions and then, these boundaries are represented through a set of features. Finally, they trained a classifier to obtain the segments and their temporal order, using the relations *before*, *after* and *incomparable*.

Li et al. [29] considered section mapping as a sequence-labeling problem to 15 possibly known section types. For training the model, they first determine text span boundaries in the training set with section headings and blank lines as cues. A text span may start with a section heading or with a blank line. The start position of the next text span becomes the marker of the end of the previous text span. Recognized section headings are mapped to section labels based on a custom dictionary. Thus, every text is represented as a sequence of text spans.

The study of Waranusast et al. [62] contributes to section identification in systems that can interpret freehand anatomical sketches, and handwritten text using temporal and spatial information. On-line digital ink documents, composed of sequences of strokes, and separated by gaps are used as input. A stroke consists of a sequence of stroke points (x and y coordinates) recorded between a pen-down event and a pen-up event. This information was used to train a classifier that distinguishes text from non-text sections, based on spatial and temporal features of the ink document. Even though the sections identified in this work are not classical clinical sections, the feature extraction methods can be extended to other types of classification.

Tepper et al. [57] used a model based on maximum entropy. They classify every text line using BIO tags with category labels X; i.e., B-X and I-X indicate that the line begin (B) or lay inside (I) a section with category X; O means the line is not in any section (e.g., a blank line at the beginning of a document).

The approach for section identification proposed by Mowery et al. [39] classifies sentences according to the SOAP framework. To this end, an SVM classifier for each SOAP class was trained. The authors found out that most of the semantic features were useless for classification, probably because they were too broad. Features identified as important for section identification were: predictive unigrams and bigrams, word/POS pairs, and some tokens to which UMLSⓇ (Unified Medical Language System) [2] identifiers had been assigned.

Lohr et al. [31] developed a custom dictionary of useful categories for the annotation of CDA (Clinical Document Architecture) [13] compliant sections and presented a guide for section annotation in German discharge summaries and related documents. They narrowed down the appropriate granularity of the annotation unit, the set of relevant and feasible categories, and their internal structure. They also highlighted text passages that do not belong to the category suggested by the subheading they were assigned to; such “out of context” text passages were abundant in their corpus and constituted a major source of false assignments.

In Deléger and Névéol [8], section identification only considered two types of sections, viz. core medical content and others, e.g., headings and footers, in order to select what is relevant for physicians. They trained a CRF model using a training data set with EHR narratives and other medical texts, e.g., email content.

Haug et al. [17] used Bayesian models to detect sections, based on training set with 98 different topics annotated in seven types of clinical narratives. The model includes different types of narratives because of the high variability in the headings produced during routine medical documentation for each kind of document. The annotations include (sub)section headings and section contents. Similar topics are labeled under the same section name.

Even though the above studies have trained their models on data sets from specific institutions, their underlying ML logic gives them the possibility to retrain the original model on new training data, in such a way that the section identification method can be applied in other institutions. Tepper et al. [57], for instance, stated that their method could be easily retrained, because it “requires only a small annotated dataset”. Nevertheless, retraining ML models, although viable in theory, is often laborious and time-consuming in practice, because new annotated data sets are required to obtain acceptable performance. Similarly, the existing methods are constrained to identify sections on certain types of clinical narratives (see Table 1); however, it is also possible to retrain the models on a new narrative type or applied the original model to narratives with similar characteristics. Mowery et al. [39] suggested their method, originally created for *Emergency Reports*, could be applied on reports with similar structure and lexical distribution, such as *History and Physical Exams*, *Progress Notes* and *Discharge Summaries*. Likewise, Bramsen et al. [3] claimed their method, originally created for *Discharge Reports*, can also be used during the pre-processing phase on other analysis.

Finally, seven hybrid approaches use rule-based methods during the creation of training and test data sets, and then apply ML methods. This is the case of Apostolova et al. [1], Sadoughi et al. [46], Ni et al. [40], Chen et al. [5], Dai et al. [7], Jancsary et al. [20], and Ganesan and Subotin [16]. Other ones use rules for detecting the explicit sections and a ML algorithm for detecting implicit sections like dine in Cho et al. [6]. These works are characterized in Tables 2, 3, and 4, and explained in detail in the following section.

#### Hybrid approaches

The study by Cho et al. [6] extracts common heading labels from a training data set and used them to identify labeled sections on the target texts. Then, it extracts patterns from the training data set and used them as features of an expectation maximization model for identifying “hidden” (i.e. unlabeled) sections. Features represent the kind of employment of the colon character (e.g., if it is used to express time, ratio or a list), and the candidate phrase characteristics (e.g., whether it is all capitalized or in title format). The model is applied when it is suspected that a text contains “hidden” sections, according to the statistics computed for the kind of text; in particular the length of a section, the number of sections and the order of sections.

In a similar vein, Apostolova et al. [1] used training data to identify common, local formatting patterns that help identify section headings and boundary markers in radiology reports. The training vectors are built using 3000 reports, computing the bi-grams weights (TF-IDF Term Frequency – Inverse Document Frequency) to the eight possible sections in radiology reports. Each sentence from the training data is assigned to one section. For new reports two classification strategies are applied: first, a rule-based one for detecting headings. When no section is detected, the similarity of the sentence to the training vectors belonging to each section is computed. This strategy assigns the sentence to the section of the closest sentence vector (cosine distance). The second strategy uses SVM for creating one classifier for each section type trained on the features of the sentence itself and of surrounding sentences. Features are sentence formatting (e.g. capitals, colon), previous sentence boundary (e.g. white space, special characters), following sentence boundary, a flag indicating exact heading matches in the sentence, and the cosine vector distance to each of the eight section vectors. Formatting and boundary features significantly improved the classification of semantically related sections such as *Findings* and *Impression*. Other sections, e.g., *Recommendation* proved to be hard to classify correctly.

Sadoughi et al. [46] embedded section identification in the processing of medical dictations, as a binary classification problem where “1” corresponds to boundary token and “0” otherwise. They used a recurrent neural network with an embedding layer using pre-trained embeddings with word2vec. They consider a continuous bag-of-words with a size of 200, trained for 15 iterations over a window of 8 words with no minimum count. We classify this study as hybrid, because they also used regular expressions.

Speech recognition was also the context of Jancsary et al. [20] approach to text segmentation. They created a data set using dictations whose completeness was validated by humans. Then, a section type was automatically assigned to each report fragment if its heading matched some known heading from a custom dictionary; when the heading was not known, they assigned it manually. Token features of each section such as dates, physical units and, dosages were labeled. Using the labeled data set, they labeled new dictations in raw format (dictations without human intervention) using a similarity function based on the semantic and phonetic differences. With both annotated data sets they built a model based on CRF for the classification of new dictations.

Chen et al. [5] and Dai et al. [7] formulated section identification as a token-based classification using CRF on lexical, syntactical, contextual, and semantic features, the latter resulting from string match against headings terms from controlled terminologies. The importance of each feature in the model was analyzed. The authors found out that adding layout features (e.g. line breaks) enabled the CRF model to recognize section headings that did not appear in the training set. A decrease in precision was observed when very specific terminology was associated with subsections in the lexicon. Finally, it was found that using non-standard abbreviations was one of the most important causes of false negatives (e.g. “All” for “allergy”).

Ganesan and Subotin [16] proposed a segmentation model that identifies header, footer, and top-level sections in clinical texts, using a regularized multi-class logistic regression model to classify each line according to five roles: start of a heading, continuation of heading, start of a section, continuation of a section, and footer. The training set was built using a rule-based approach applied to different kinds of clinical narratives. Using this training set, the method generates features at text line level, including the relative position of the line, which turned out to be the most important indicator for section boundaries. Another useful feature is the “KnownHeading” feature that represents whether the line contains a heading included in a custom dictionary.

Ni et al. [40] used active learning and distant supervision during the creation of the training data set for a section identification maximum entropy model. Active learning reduces annotation effort by selecting the most informative data, and distant supervision automatically generates “weakly” labeled training data using a knowledge base. The study starts with a manually annotated training set, which is then used to predict the section on a new data set. From the predicted data set, the examples with the lower prediction confidence are selected and given to a human annotator. The confidence of the examples (i.e. documents) is calculated by counting the sentences that have a confidence less than 0.9 and selecting the top m documents using this count. In the distant learning approach, the goal is to include all the known medical headings in a custom dictionary and use them for extracting, from the unlabeled data set, the sentences that contain them. The found heading is used to tag the sentence and all the subsequent sentences until another heading is found. This study demonstrated that both techniques achieved a good accuracy on the final training data set and the models generated from it.

### Application scenarios

In many NLP projects, section identification is just one task that is expected to enhance the accuracy of other tasks like information extraction. Such application scenarios are elucidated in Table 5. The assumption is that knowing the specific position of a concept in the clinical narrative allows more context to be given to the task to be performed.

**Table 5:** Application scenarios

Application	Studies
Building structured data	Rubin and Desser [45], Mowery et al. [39], Kropf et al. [25]
Contextualized search	Meystre and Haug [36], Wang et al. [60], Doan et al. [12], Schuemie et al. [50], Lin et al. [30], Shivade et al. [51], Mehrabi et al. [34], Singh et al. [52]
Coreference resolution	Xu et al. [65], Lei et al. [28]
Named entity recognition	Lei et al. [28]
De-identification process	Phuong and Chau [42]
Temporal analysis	Bramsen et al. [3], Lee and Choi [27]
Education	Denny et al. [11]
Quality analysis	Hsu et al. [18], Sadoughi et al. [46], Melton et al. [35]

Section identification enhances unstructured text by a rough. That is why some studies in EHR have used the detected sections to build structured data repositories or robust structured resources that can be easily queried and analyzed by an institution or shared among researchers [25, 39, 45]. While the above application is the most obvious, the most common is to use sections to contextualize the search for relevant information about a patient. Identifying the sections is useful to **focus** the search on a specific section or the opposite, **to augment** the number of sections where the search should be applied.

Doan et al. [12] used section identification to improve the precision of medication extraction, by removing irrelevant drug mentions occurring in the allergy, lab, or study sections. They argued that medication search had to be focused on specific sections. Similarly, Lin et al. [30] used sections for the automatic identification of methotrexate-induced liver toxicity in patients with rheumatoid arthritis. They improved their F1 score when focusing only on highly informative and relevant sections. It was also used for prioritizing clinical data through the extraction of the *Impression* section from radiology reports [52]. Besides, the study of Shivade et al. [51] improved the precision when analyzing risk factors for heart disease among diabetic patients including rules that consider the section where a term is found. Similarly, Edinger et al. [14] improved cohort selection by reducing the number of sections analyzed in order to retrieve a specific patient characteristic. Mehrabi et al. [34] improved the detection of family history facts outside the family history section. Similarly, Schuemie et al. [50] used sections for clinical query expansion and demonstrated that some sections are more informative regarding disease concepts, *viz.* the sections postoperative diagnosis, chief complaint, diagnosis in discharge summaries. Wang et al. [60] used contextual filters for detecting “disease-manifestation related symptom” and “drug-adverse drug event types” relations. The filters may be used to focus the analysis on sections or section groups. They demonstrated that recall and precision were improved in detecting specific relations of interest. Likewise, Meystre and Haug [36] extracted sections and used them as the context for recognizing medical problems that contributed to populating a problem list. Besides searching for clinical information, Phuong and Chau [42] used section identification for improving EHR de-identification. They demonstrated a positive impact on de-identifying age, location and phone number. Co-reference resolution and named entity recognition are other tasks for which a benefit of section identification could be shown. According to Xu et al. [65], co-reference resolution for Person, Problem/Treatment/Test and nouns in EHR could be enhanced by using the section as an input feature for the classifiers. Furthermore, Lei et al. [28] proved that their named entity recognition strategy achieved the highest performance by combining word segmentation and section information. Temporal analysis is another relevant application of section identification. The goal is to identify sections as a means to detect temporal changes in a clinical trajectory. The study of Bramsen et al. [3] and Lee and Choi [27] followed this approach, obtaining good results in terms of precision and recall.

Finally, section identification had been used for education and quality assurance. Denny et al. [11] e.g., used it in an automated education advisor system that analyzed students’ notes for relevance, regarding two geriatric competencies. The system identified UMLSⓇ concepts in sections of student’s notes. According to these concepts, the system generated custom email alerts with supplemental learning material customized to students’ notes. From a quality perspective, Hsu et al. [18] identified specific sections of radiology and pathology reports in order to perform automated quality assessment of radiology interpretations. Their goal was to extract the diagnosis from a patient’s radiology report and compared it with the pathology report. For doing so, they had to extract the diagnosis only from relevant sections. In Sadoughi et al. [46] post-processing quality analysis of medical dictation was improved by using real-time section identification. Likewise, Melton et al. [35] analyzed the real use of controlled terminology in clinical texts, using the detected sections. They concluded that 20% of the sections did not map to any of the terms of the analyzed standards.

### Section identification performance metrics

This section focuses on the performance metrics, reported by proponents of section identification approaches. Such metrics varied greatly according to the test data sets used during their experiments. Regarding the metrics, some of them discriminated performance for labeled and unlabeled sections, while others reported one measure, regardless of section type. Likewise, some studies aimed at accuracy only, while others specified precision, recall, and f-measure. Observing the number of measures reported, some studies included a huge number of experiments, and sometimes, presented their results not as an absolute value, but as a range of values (e.g. precision range). Other studies did not report any performance measure, especially when rule-based methods alone were employed. From an experimental point of view, the majority tested their methods using data sets obtained from the same data source from which the training data was extracted. This implies that most of them have not been tested to be useful in different institutions, except one hybrid method aiming at evaluating its adaptability by using data from several institutions [16].

Considering these performance result differences and being aware of the difficulties in comparing experimental results, we examined the performance reported for each study and extracted precision, recall, F-measure or accuracy, when available. For studies reporting more than one experiment, we took their best result. For those that reported their performance results using a numerical range or reported individual results for each class value, we calculated the average.

**Fig. 2:**
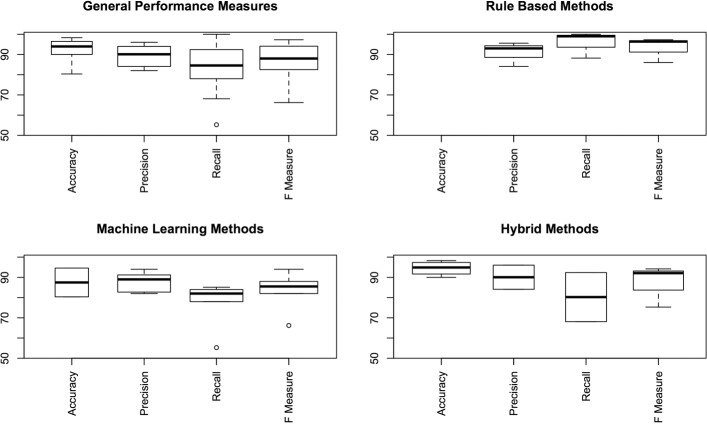
Distribution of performance results

**Fig. 3:**
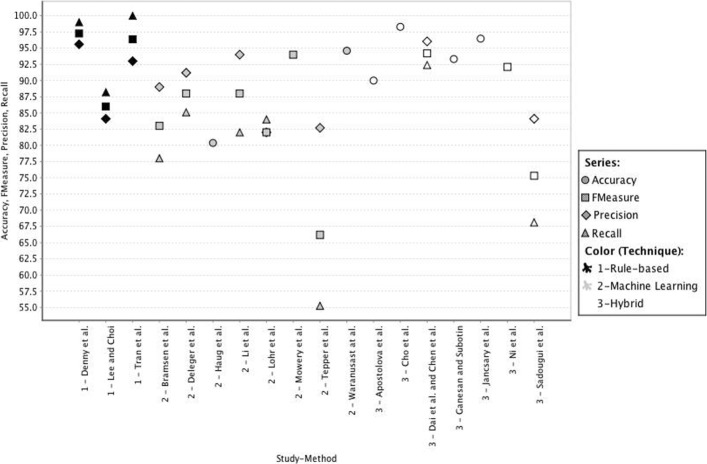
Individual performance results

Figure 2 shows the distribution of performance results and Fig. 3 illustrates the individual results. In general, accuracy and precision were very high (i.e. Accuracy median =93.96*%*, Precision median =90.1*%*), whereas the recall was in general lower (i.e. median =84.55*%*). Contrary to the intuition, the recall from rule based methods was better than the recall using ML and hybrid methods.

Only three out of 23 studies reporting on a rule-based method published results [10, 27, 58], which does not allow us to generalize their good results. Besides, the experimental scenario of Tran et al. [58] was focused on the extraction of the *Family history* section (precision =93*%* and recall =100*%*). Such a specific result cannot be compared to the experiments able to extract any section. Nonetheless, the rule-based method proposed by Denny et al. [10], is one of the most cited and complete methods, which achieved very good results (precision =95.6*%*, recall =99*%*) in the analysis of “history and physical” notes.

From the group of studies that used ML methods (i.e. eight studies), the highest performance was achieved by Li et al. [29] (i.e Accuracy =94*%*), by Waranusast et al. [62] (i.e. accuracy =94.6*%*) and by Mowery et al. [39] (FScore =94*%*), the first one using a hidden Markov model and the others a SVM model. However, their experimental setups are not comparable; e.g., the experiments presented in Mowery et al. [39] included only 50 records and performed a CV approach, while Li et al. [29] used 2130 records for testing using a hold out validation approach, and the experiments in Waranusast et al. [62] used a CV approach with 10694 records (see Table 3).

In general, the recall in the ML group is the lowest. It can be argued that this is the rationale for combining ML models with rules. In fact, the hybrid methods under scrutiny achieved better performance results than methods based on ML alone. The best scoring ones are the methods proposed by Cho et al. [6] (accuracy oscillating between 97.2% and 99.4%) and by Jancsary et al. [20] (accuracy =96.48*%*), who used an expectation maximization classifier and a CRF model, respectively. However, the experiments reported by Cho et al. [6] did not specify the characteristics of the validation set. On the contrary, the work of Jancsary et al. [20] reported a hold out validation approach using 1003 records in the test set. Aside from these studies, the set of experiments presented by Ganesan and Subotin [16] are very valuable; their logistic regression model obtained good results (accuracy =93.32*%*) with different types of texts from different institutions. They reported experiments with 12502 records using a hold out validation approach. This is the only study that sought flexibility in its proposal.

### Terminology systems

Several section identification methods rely on section headings or semantic annotations of section text, rooted in custom dictionaries or controlled terminologies. The majority of studies created custom dictionaries (28 studies), ranging from term lists, hierarchies to ontologies. The most common controlled terminology used is UMLSⓇ [2] in ten studies, followed by LOINCⓇ [33] in eight, Meystre&Haug dictionary [36] in seven, SecTag [9] and QMR (Quick Medical Reference) [38] in six, HL7-CDA [13] in three, and both the Consumer Health Vocabulary [59] and Wordnet [37] in one study.

In section identification UMLSⓇ has been used to parse and transform narratives into structured representations consisting of codes [20, 39, 49, 60]. It has also been used for the creation of specific terminologies for section identification, as SecTag [9]. LOINCⓇ is included in UMLSⓇ; therefore some studies using UMLSⓇ may implicitly use LOINCⓇ, among others. Besides, LOINCⓇ was originally conceived as a vocabulary for observations in HL7 messages; therefore, it is also implicitly used in the studies that referred to the HL7-CDA standard. SecTag, the terminology of the SecTag system is a reference and interface terminology for clinical section headings that integrated other terminologies and expanded them [9]. The integrated terminologies are QMR terminology, LOINCⓇ, and a custom dictionary with 559 headings created by Meystre and Haug [36]. To interoperate with other systems, SecTag retained the identifiers used in LOINCⓇ and in UMLSⓇ. Since several studies used the SecTag system (or an adaptation), these studies implicitly used all the terminologies contained in it.

HL7-CDA is a standard that describes the structure and semantics of a clinical document for the purpose of exchange [13]. The identifiers of the clinical document sections generally come from LOINCⓇ. It has been used to represent operative note section headings and to develop a resource for operative note section headings [35]. Lohr et al. [31] used the German version of the HL7-CDA for the annotation of sections in discharge summaries and related documents. Additionally, Haug et al. [17] used this standard to analyze whether the sections obtained using their method were CDA-compliant.

The “Consumer Health Vocabulary” is also included in the UMLSⓇ, and its purpose is to connect informal, common words and phrases about health to technical terms used by health care professionals [59]. Shivade et al. [51] used it to identify candidate annotations that indicate risks of heart disease in clinical sections.

Finally, Wordnet, a lexicon mainly for the English language, was used in the method proposed by Jancsary et al. [20] for detecting semantically similar words. Its content, together with UMLSⓇ, was used to semantically compare the words available in annotated narratives with the words in new narratives. For each semantic match, the annotation was assigned to the new token.

## Discussion

This review of the state of the art in section identification in clinical narratives is the first in-depth review of the methods for structuring clinical narratives through section identification and annotation, comprising 39 studies (37 unique studies).

No more than half of the studies were based on formal methods, whereas the other ones used or adapted previously created or proposed approaches that addressed their specific needs, but were not tested in other contexts. Less than one fifth operated languages other than English. The assumption that sections have headings was made in nearly half of the works. This assumption is problematic because sections without headings are very common (two thirds according to [29]). Although some institutions and jurisdictions have defined precise templates for clinical narratives, these are often disregarded. E.g., [56] showed that *Treatment* and *Recommendations* are sometimes merged, as well as *Anamnesis* and *Medical and risk factors*, and sections are omitted when considered irrelevant, e.g., *Medical comments*. Besides, they also found that some sections were domain dependent, e.g., *Family medical history* had only been included when relatives had been diagnosed with a relevant disease.

In the studies under examination, very little information was available regarding text production details or the exact source of the texts. It is sensible to assume that the more freedom a clinical author has, the less standardized section headings can be expected and the more often sections without heading will be found. In contrast, where clinical texts obey immutable, pre-defined document templates, no NLP methods for section identification are needed.

There are different granularity levels under scrutiny. Whereas some studies considered classical dissections of clinical documents according to templates like SOAP, others dissected texts according to the temporal changes referred to in the text, and others just distinguished clinically relevant content from institutional/administrative information such as found in headers and footers.

Regarding the technologies employed, three groups are distinguished: rule-based, ML and hybrid. Most rule-based approaches require sections with headings, which are processed using string and pattern matching. Others, using probabilistic rules based on text features, produced very good results. ML methods treat section identification as a classification problem, with CRF and SVM being the most popular paradigms. A bottleneck is the time-consuming production of training data, with the drawback that the models are difficult to adapt to other contexts. Typical training features were lexical, syntactical, semantic or contextual ones. Finally, hybrid approaches used rule-based methods, often during the creation of training data sets, and then applied ML methods.

The predictive value of the features used by some of the ML and hybrid studies revealed remarkable phenomena. E.g., features like token unigrams and n-grams, word/POS pairs, features indicating whether the text contains a known section heading, and layout features had a substantial positive impact on the classification. In addition, the use of non-standard abbreviations in section headings and the appearance of phrases in sections that do not correspond constituted major reasons of incorrect classifications. Similarly, a decrease in precision on the section identification was observed when a very specific terminology for sub-headings was used.

Almost all the studies relied on a custom dictionary; mostly constructed by the authors themselves, occasionally re-using content from existing controlled terminologies. Here, SecTag, an extract of several terminologies is gaining popularity [9]. The most common source of external terminology is the UMLSⓇ metathesaurus, but most studies did not specify which UMLSⓇ source vocabularies they actually used.

The contexts where section identification has been used was analyzed considering the specific application and the involved type of narrative. Application contexts are diverse; ranging from the generation of structured data for analytical purposes to the transfer of documents to external parties. Information retrieval of EHR plays a significant role like the support of cohort selection or the identification of patients with risk factors. There is also experimental evidence that co-reference resolution and named entity recognition are improved when using the section as another feature of the model. Sections have also been useful for disambiguating whether a text token is a sensitive entity in EHR de-identification tasks. Likewise, the identification of temporal sections on clinical narratives has been applied to improve tasks that bring events in a specific order. Section identification was also used to support the quality assessment of clinical texts, e.g., for measuring their level of compliance with institutional guidelines. Similarly, section identification has been used to extract relevant concepts from texts produced by students in order to select supportive educational resources for the specific topic students were coping with.

Regarding the text types under scrutiny, discharge reports constitute the leading text genre, followed by radiology reports, visit notes, and pathology reports in decreasing order. The large number of possible notes reveals the lack of a standardized typology of clinical narratives.

Performance analysis showed that most reported results are incomparable due to the absence of any reference standard for section identification evaluation. Although often viewed as a classification task, the level of granularity of the classification largely varies from studies that focused on the token or sentence level, to studies that focused on section level.

Aware of these limitations, we examined the reported measures in each study and found that precision and recall are high. However, recall is always lower in the reported studies. We also found that the more restricted the scope in terms of text genre, medical specialty or institution the better the results. Regarding the kind of method, we found that most of the studies with rule-based methods did not report any kind of evaluation measure, but the ones that reported obtained very good results. Hybrid methods got better results than ML methods, but lower than rule-based methods. However, their scope was more ambitious than rule-based studies in terms of the type of notes they can deal with. The lack of large data sets for training and for testing is one of the major difficulties in all the studies that used a ML method.

There are several limitations to this review. First, it may have missed relevant articles that were not indexed in the selected databases. Second, though the definition of the keywords involved much work and several iterations, we could have missed some studies that used less common language terms to refer to section identification or which were written in other languages. Finally, given the nature of this review, viz. an overview of academic literature, we are aware that much work done in the development of industrial products is not reflected. Neither did we analyzed patents related to section identification approaches that are not paralleled by academic papers.

## Conclusion

We have reviewed and characterized current methods for identifying sections in clinical narratives from EHRs, by analyzing 39 studies. There is clear evidence that they improved the understanding of clinical narratives, which has a positive impact on the performance of different NLP tasks. Thus we recommend that section identification should be included in NLP pipelines that handle unstructured clinical narratives.

For clinical NLP in general and for section detection in particular, a major challenge is the lack of pre-existing annotated corpora. One reason for this is data privacy. The effort needed for clinical document de-identification and the often unclear legal status of de-identified clinical documents for research explains this situation. Besides, it is unclear to which extent a certain de-identification protocol can prevent re-identification, especially for some type of documents (e.g. discharge summaries).

To deal with this problem, a classic solution would be to use rules that embody the characteristics of the sections. However, this solution reduces the generalizability to other narratives from the same type, but from different authors and institutions. Combining supervised with unsupervised ML methods that analyzed semantic similarities of known and unknown EHR structures may be a good path to create more stable solutions; unfortunately, this path has not yet explored in depth for section identification.

Reported evaluation experiments of current section identification methods may be improved with an evaluation framework that defines measures enabling result comparison, independently of the proposed method, and that includes an open benchmark data set. The challenge for the community is then to produce this benchmark data set, integrating narratives from different institutions, and agree on the type of measures to be evaluated. This should be done for different natural languages.

Another unexplored issue is the automatic recognition of the conceptual model behind the clinical narratives; there are significant differences in the assumptions practitioners make when dealing with clinical data on a timeline in chronological order than when dealing with problem-oriented EHR, or source-oriented EHR, where texts are organized according to the method by which the information was obtained. Dealing with these differences in the written approach is a challenge that has to be fulfilled to create more generalized solutions. The interdependencies between EHR paradigms and workflows on the one hand and clinical text, on the other hand, requires further investigation.

The analysis of the applications allowed us to detect that when a method of section identification is used within a broader task, generally the simplest methods with low recall are used. The challenge, therefore, is to use the most robust methods and integrate them into high-impact clinical NLP tasks such as clinical entity recognition, acronym or abbreviation expansion, and patient record summarization.

Finally, we would like to state that in this review, we have focused on the analysis of methods published in research papers available in the selected scientific databases. It is necessary to explore other solutions available in industry-software or patents, which may lead to other findings. We also anticipate that the current and future work on section identification is being strongly influenced by the current paradigm shift towards neural network-based high-performance computing, which may affect the relevance of some of the approaches analyzed in this review.

## Data Availability

Not applicable.

## Abbreviations

APIE: Assessment, plan, implementation, and evaluation
CDA: Clinical document architecture
CV: Cross validation
CRF: Conditional random fields
CSV: Comma-separated values
EHR: Electronic health record
EMR: Electronic medical record
IC: Inclusion criteria
ML: Machine learning
NLP: Natural language processing
POS: Part of speech
SOAP: Subjective, objective, assessment, plan
SVM: Support vector machine
TF-IDF: Term frequency – inverse document frequency
UMLSⓇ: Unified medical language system
